# Ten years of delirium management: Evolving psychopharmacological practices in a tertiary hospital

**DOI:** 10.1192/j.eurpsy.2026.12015

**Published:** 2026-07-31

**Authors:** D. Üzüm, A. T. Altunç, M. E. Boylu, B. Ç. Poyraz, S. Turan

**Affiliations:** Department of Psychiatry, Istanbul University - Cerrahpasa, Cerrahpasa Medical School, İstanbul, Türkiye

## Abstract

**Introduction:**

Management of delirium involves pharmacological interventions for symptoms such as agitation, restlessness, insomnia, and psychotic features. However, preferences for psychotropic medication are evolving. This change is caused by new clinical guidelines, new drugs, and a greater focus on patient safety. In this study, we examined the changing trends in the management of delirium in our clinic over the past decade.

**Objectives:**

To investigate the changing trends in delirium management.

**Methods:**

This retrospective study analysed psychiatric consultation notes of the patients referred for suspected delirium. For comparative purposes, we included consultation notes from two spesific three-year periods: August 2016 - August 2019 and June 2022 - June 2025. A total of 1,812 consultation notes were examined, and 1,657 cases confirmed as delirium were included. Data were obtained from the hospital’s information management system. For both periods, we noted the recommended psychotropic medications, and statistically analysed the type and frequency of these recommendations. Descriptive statistics were used to analyse the data. We utilised the chi-square test for comparisons between groups and, where appropriate, other parametric or non-parametric tests. All statistical analyses were performed using SPSS (IBM SPSS Statistics, Version 26).

**Results:**

A total of 1,657 consultations for suspected delirium were evaluated, including 755 cases between 2016–2019 and 902 cases between 2022–2025. The frequency of cases receiving no psychotropic medication increased significantly from 2.9% to 8.3% (p = 0.001). The use of haloperidol decreased from 34.2% to 16.5% (p < 0.001), and quetiapine use also declined from 59.7% to 48.9% (p = 0.001). In contrast, olanzapine prescriptions increased substantially from 0.7% to 22.0% (p < 0.001). The use of melatonin or melatonin agonists also rose significantly (0.7% vs. 2.9%, p = 0.002). No significant differences were observed in the use of risperidone, other antipsychotics, atypical antidepressants (mirtazapine, mianserin, trazodon), or benzodiazepines across the two periods.

**Image 1:**
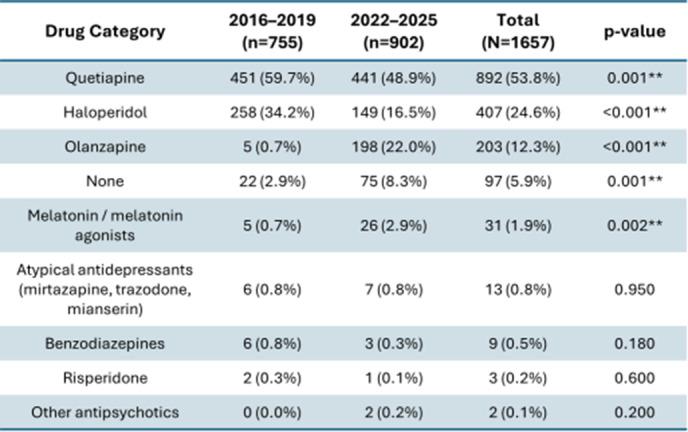
Image 1: Long description.

**Conclusions:**

With this study, it was observed that quetiapine has been the most frequently preferred psychotropic in the treatment of delirium throughout years. After comparing two periods, there is a shift away from typical antipsychotics (haloperidol). An atypical antipsychotic, olanzapine, has become a more recommended medication. Melatonin and melatonin agonists have been preferred more frequently as a supportive treatment. It is also noteworthy that there is an increased tendency not to recommend pharmacotherapy, especially for patients with hypoactive delirium.

**Disclosure of Interest:**

None Declared

## Long descriptions

### Image 1: Long description

The table consists of five columns: Drug Category, 2016 to 2019 (n equals 755), 2022 to 2025 (n equals 902), Total (N equals 1657), and p-value.

* Quetiapine: 451 (59.7%) in 2016 to 2019; 441 (48.9%) in 2022 to 2025; Total 892 (53.8%); p-value 0.001 double asterisk.

* Haloperidol: 258 (34.2%) in 2016 to 2019; 149 (16.5%) in 2022 to 2025; Total 407 (24.6%); p-value less than 0.001 double asterisk.

* Olanzapine: 5 (0.7%) in 2016 to 2019; 198 (22.0%) in 2022 to 2025; Total 203 (12.3%); p-value less than 0.001 double asterisk.

* None: 22 (2.9%) in 2016 to 2019; 75 (8.3%) in 2022 to 2025; Total 97 (5.9%); p-value 0.001 double asterisk.

* Melatonin / melatonin agonists: 5 (0.7%) in 2016 to 2019; 26 (2.9%) in 2022 to 2025; Total 31 (1.9%); p-value 0.002 double asterisk.

* Atypical antidepressants (mirtazapine, trazodone, mianserin): 6 (0.8%) in 2016 to 2019; 7 (0.8%) in 2022 to 2025; Total 13 (0.8%); p-value 0.950.

* Benzodiazepines: 6 (0.8%) in 2016 to 2019; 3 (0.3%) in 2022 to 2025; Total 9 (0.5%); p-value 0.180.

* Risperidone: 2 (0.3%) in 2016 to 2019; 1 (0.1%) in 2022 to 2025; Total 3 (0.2%); p-value 0.600.

* Other antipsychotics: 0 (0.0%) in 2016 to 2019; 2 (0.2%) in 2022 to 2025; Total 2 (0.1%); p-value 0.200.

Navigate back to Image 1:.

